# The Role of *WWOX* Gene Variant in Hypospadias and 46,XY Disorders of Sexual Development

**DOI:** 10.1007/s43032-026-02112-9

**Published:** 2026-05-04

**Authors:** Yasemin Denkboy Ongen, Havva Tezcan-Unlu, Ufuk Unal, Ecem Efendi-Erdem, Gulsah Cecener, Erdal Eren

**Affiliations:** 1 Department of Pediatric Endocrinology, Faculty of Medicine, Bursa Uludag University, Bursa, Türkiye; 2 Department of Medical Biology, Faculty of Medicine, Bursa Uludag University, Bursa, Türkiye

**Keywords:** Disorders of sexual development, Hypospadias, Genomic DNA, Mutation, Protein analysis, *WWOX* gene

## Abstract

46,XY disorders of sexual development (46,XY DSD) are conditions characterized by deviations from typical male sex development, which encompasses a broad spectrum of clinical features such as hypospadias, decreased sperm production, dysgenetic testes, bifid scrotum, and the presence of a mature uterus and fallopian tubes. The *WWOX* gene, which has been implicated in various cancers, is also thought to play a role in sex development, although its involvement in 46,XY DSD remains poorly understood. This study aimed to investigate the *WWOX* gene in a patient presenting with hypospadias. A 7-month-old male with a history of in-vitro fertilization pregnancy due to male factor and premature birth was referred to our clinic for hypospadias and undescended testicles. The physical examination showed scrotal hypospadias and significant chordee. Chromosome analysis was 46,XY, and the sex-determining region Y protein was positive. Using DNA sequence analysis and protein expression studies, the p.Ala141Thr variant in the *WWOX* gene was identified. DNA sequencing analysis revealed that the patient is homozygous for this variant, with the father being homozygous (having an infertility history but no physical examination) and the mother heterozygous for the same mutation. Western blot analysis revealed significantly reduced WWOX protein levels in both the proband and father compared to the healthy controls, indicating that the variant impaired protein expression. These findings were consistent with the results from in-silico analyses, which predicted that the p.Ala141Thr substitution disrupts the secondary structure of the WWOX protein, suggesting a functional impact on its activity.

## Introduction

46,XY disorders of sexual development (DSD) (46,XY DSD) refers to a group of conditions in which individuals with an XY chromosomal pattern fail to develop typical male genitalia [1]. A critical factor in sex determination is the complex interplay between testicular and ovarian signaling pathways, which are regulated by precise gene expression (2). Even minor alterations in the dosage of key genes can disrupt this balance, leading to DSD [3]. This disorder is commonly associated with mutations in the *SRY* gene on the Y chromosome, or in other genes involved in sex differentiation, such as *SOX9, DHH*, and *NR5A1* [1]. The clinical manifestations of 46,XY DSD vary widely, ranging from mild hypospadias to more severe cases involving ambiguous genitalia and internal reproductive organs resembling those of a female [2].

One gene of interest in this context is *WWOX* (WW domain-containing oxidoreductase), a tumor suppressor gene located on chromosome 16q23.1-q23.2. *WWOX* plays a crucial role in various cellular processes, including differentiation, apoptosis, and cell cycle regulation [4]. Its expression is particularly high in endocrine organs, including the testis and ovary [5], which are key tissues involved in sex differentiation and steroidogenesis. However, the role of *WWOX* in 46,XY DSD remains largely unexplored.

Recent studies have highlighted the potential involvement of *WWOX* mutations in developmental disorders, including their association with gonadal development and steroid metabolism [5–7].

The objective of this study was to investigate potential mutations in the *WWOX* gene in a patient diagnosed with hypospadias, a common clinical feature of 46,XY DSD. Notably, a homozygous variant (c.421G > A, p.Ala141Thr) in the fifth exon of *WWOX* was identified in the patient.

## Case Presentation

A 7-month-old male was referred to our clinic for hypospadias. Hypospadias and undescended testicles were noted from birth. The patient, who had no additional endocrinological complaints, was evaluated with a weight, height and head circumference below the 3rd percentile, the right testicle about 1 cc in the scrotum, the left testicle about 1 cc in the inguinal canal, the stretched penis length of 2 cm (< −2 SDS), scrotal hypospadias, and significant chordee (congenital penile curvature) in physical examination. The patient was born to a 36-year-old G1P1 preeclamptic mother at 29 + 5 weeks of gestation with an APGAR score of 5–8 and a weight of 975 g, via cesarean section from an in vitro fertilization (IVF) pregnancy, and was admitted to the neonatology intensive care unit due to prematurity and respiratory distress. He received intensive care for 2 months and was discharged weighing 2500 g. There was no consanguineous marriage or similar family history.

Laboratory tests at an external center revealed FSH levels of 1.9 IU/mL, LH levels of 5.38 IU/mL, total testosterone of 1.69 μg/dL, DHEAS of 1668 μg/dL (60–493 μg/dL), and 17-OH progesterone of 0.559 ng/mL, at the age of one month old. Tests performed at our clinic revealed FSH levels of 1.41 IU/ml, LH levels of 1.05 IU/ml, total testosterone of 1.12 μg/L, and DHEAS levels of 48 μg/dL. Pelvic ultrasound imaging showed no uterus, and the testicles were seen in the bilateral inguinal canals. Chromosome analysis was found to be 46,XY, and the sex-determining region Y protein (SRY) was positive. To assess the patient’s testosterone response, he was given low-dose intramuscular testosterone therapy (25 mg/month) for 3 months, and at follow-up, a stretched penile length of 3 cm was measured, interpreted as a partial response.

## Materials and Methods

### Ethics Statement

This research was conducted in accordance with the principles of the Declaration of Helsinki and received approval from the University Ethics Committee (Approval No: 2011-KAEK-26). All procedures were performed in compliance with the approved ethical standards. Written informed consent was obtained from all participants prior to their inclusion in the study.

### Sample Collection and DNA Isolation

In this study, DNA samples were isolated from the peripheral blood of the patient and their parents, as well as from 100 healthy pediatric controls. Peripheral blood DNA isolation was performed using the Trizol method. The quality and quantity of the isolated DNA samples were measured using a UV–Vis Spectrophotometer (Beckman Coulter, USA). Only samples with an A260/A280 ratio ranging from 1.7 to 2.0 were included in the study.

### Polymerase Chain Reaction and Sanger Sequencing

Primers specific to the *WWOX* gene were designed using the platform available at https://www.ncbi.nlm.nih.gov/tools/primer-blast/.

The designed primers were forward primer (F: AAGTGGTGCTCCGGTAAAGG) and reverse primer (R: AGCCCGGCATGTGTATTTGA), with an amplification product length of 246 bp. PCR was performed using genomic DNA extracted from the patients’ peripheral blood, along with the relevant primers. The PCR reaction was performed in a total volume of 10 µL, comprising the following components: 50–100 ng of genomic DNA, 1.25 units of Taq polymerase, 0.8 mM of each dNTP, 1.5 mM MgCl2, and 0.5 µM of each primer. PCR products were assessed using 2% agarose gel electrophoresis.

For mutation analysis of the *WWOX* gene, the obtained high-quality PCR products were purified using the PCR Clean-Up System. Separate sequencing reactions for the forward and reverse primers were carried out using the DTCS Quick Start Kit (Beckman Coulter Inc., California, USA). After alcohol washes, the purified products were treated with Sample Loading Solution to prepare them for sequencing, followed by sequencing analysis using the Automated Sequencing System (Beckman Coulter Inc., California, USA). The resulting nucleotide sequences were compared with the reference sequence of the relevant exon of the *WWOX* gene, available in the Ensembl database (http://www.ensembl.org/index.html), to identify potential mutations.

### Western Blot Analysis

The effect of the identified *WWOX* gene mutation on protein levels was examined through Western blot analysis. After preparing the total cell lysate, protein concentrations were determined using a Qubit device, and equal amounts of protein (µg/mL) from each sample were denatured with SDS-sample buffer by heating at 95ºC for 5 min. Denatured protein samples were loaded into a 4–20% polyacrylamide gel in equal amounts for each well and subjected to electrophoresis at 110 V for 2 h using a BioRad Mini-Protean device. Subsequently, the protein bands were transferred onto a PVDF membrane at 4 °C for 2 h. After the transfer, the membrane was blocked for at least 1 h at room temperature with non-fat milk prepared in 1X TBS-Tween 20 solution, followed by overnight incubation with appropriate primary antibodies diluted in 2% BSA. To remove unbound antibodies and reduce nonspecific binding, the membrane was washed three times for 10 min each with TBS-T solution, and then incubated with suitable secondary antibodies for 1–2 h at room temperature. Finally, the membrane was washed with 1X TBS-T, and chemiluminescent imaging was performed using the Pierce kit. WWOX protein was detected using the WWOX Protein Antibody (CST 4045S) and ß-Actin (13E5) Rabbit Monoclonal Antibody (Cell Signaling Technology, Danvers, MA) as a loading control for normalization of protein loading, and the blots were imaged using the C-DiGit® Blot Scanner (LI-COR Biosciences, Lincoln, NE).

## Results

**Fig. 1:**
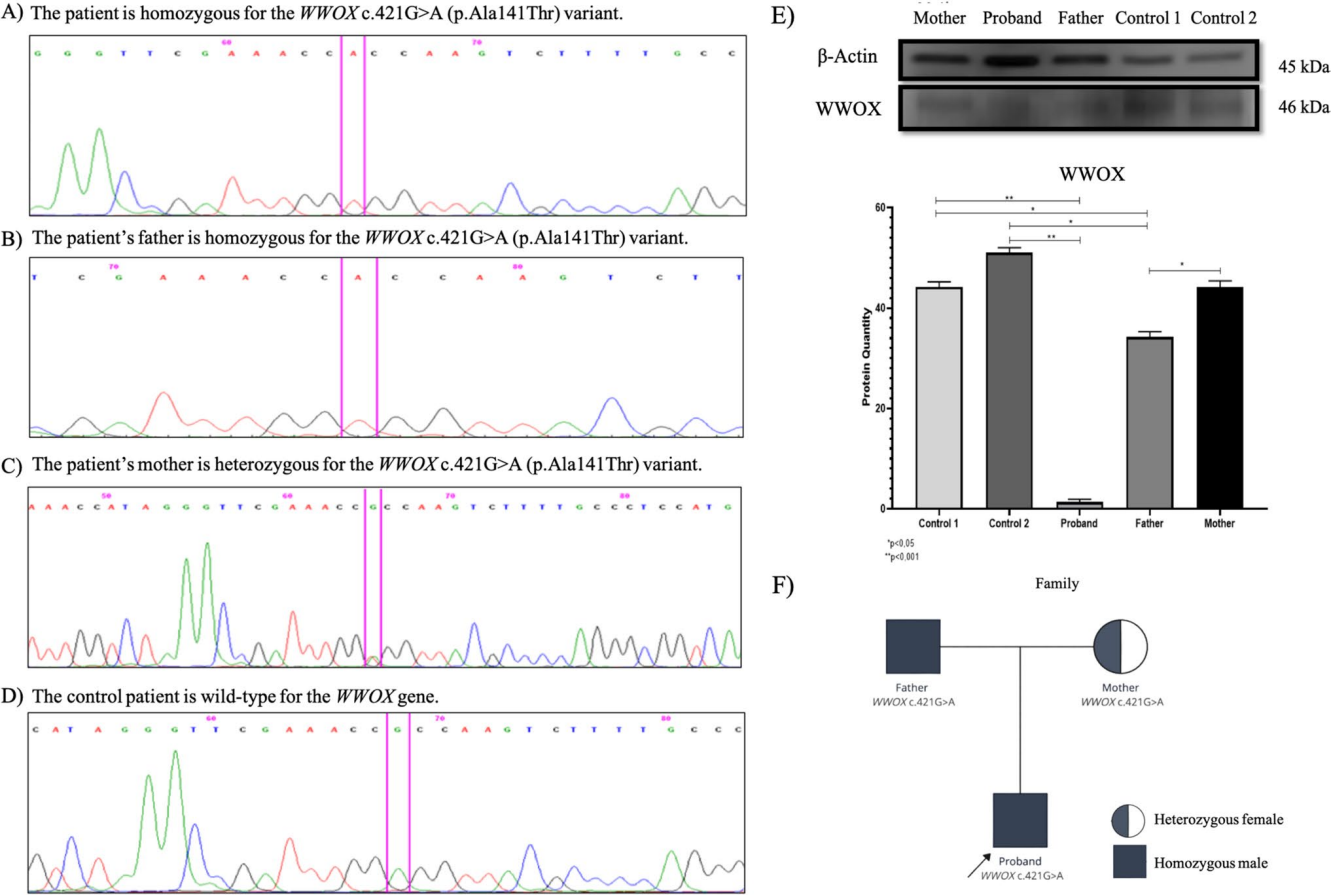
The figure illustrates the results of DNA sequence analysis from a family carrying the *WWOX* c.421G > A (p.Ala141Thr) variant (**A**) demonstrates the proband is homozygous for the mutation, (**B**) shows the father is homozygous, and (**C**) indicates the mother is heterozygous. (**D**) presents the wild-type sequence in a control individual. (**E**) Western blot analysis reveals significantly reduced WWOX protein levels in the proband and father compared to controls, with β-Actin used as the loading control. (**F**) The family pedigree outlines the inheritance pattern, where the father is homozygous, the mother is heterozygous, and the proband is a homozygous male. Statistical analysis shows significant differences in protein levels (p < 0.05 and p < 0.001)

In this study, a *WWOX* c.421G > A (p.Ala141Thr) mutation was identified in a patient diagnosed with hypospadias, a common feature of 46,XY DSD. This mutation was absent in 100 healthy pediatric controls, suggesting its potential pathogenicity. DNA sequencing analysis revealed that the patient is homozygous for the *WWOX* c.421G > A variant, with the father being homozygous and the mother heterozygous for the same mutation. The control sample exhibited a wild-type sequence, further validating the presence of this mutation in the affected family members. Western blot analysis revealed significantly reduced WWOX protein levels in both the proband and the father compared to healthy controls, indicating that the mutation results in impaired protein expression. These findings were consistent with the results from *in-silico* analyses, which predicted that the p.Ala141Thr substitution disrupts the secondary structure of the WWOX protein, suggesting a functional impact on its activity (Fig. 1). The *in-silico* prediction tools, PolyPhen and SIFT, classified the variant as probably damaging and tolerated, respectively. The allele frequency in the general population was found to be very low (2.04 × 10⁻^4^). ClinVar lists the variant with conflicting interpretations of pathogenicity, including uncertain significance, benign, and likely benign. However, the mutation was classified as disease-causing in the MutationTaster database, providing additional support for its potential role in disease development (Table 1). Taken together, these findings suggest that the *WWOX* c.421G > A (p.Ala141Thr) variant may contribute to the pathogenesis of 46,XY DSD, particularly in relation to hypospadias. The father was diagnosed with the variant, but he refused a genital examination and verbally stated that his testicles and penis were normal. However, an IVF pregnancy, the absence of a uterine-ovarian problem in the mother, and the consideration of a male factor suggest 46XY DSD.

**Table 1:** Results of *in-silico* analysis for *WWOX* variant

Nucleotide change	Transkript	Rs number	Protein change	Polyphen/SIFT	GnomAD	Mutation t@sting prediction	Ensembl	ClinVar
*WWOX* c.421G > A	ENST00000566780.6	rs369907002	p.Ala141Thr	Probably damaging: 0.999 (sensitivity: 0.14; specificity: 0.99)/Tolerated (0.080)	Total, Allele number: 1613908 Allele frequency: 2.04e-4	Deleterious	Missense variant	Conflicting classifications of pathogenicity Uncertain significance; Benign; Likely benign

Further studies, involving larger cohorts and functional assays, are required to confirm the pathogenic role of this mutation and to explore its implications for gonadal development and sex differentiation.

## Discussion

*WWOX*, a gene with tumor suppressor activity, is involved in various cellular processes, particularly in the regulation of apoptosis and cell differentiation. Its high expression in gonadal tissues such as the testis and ovary suggests that it may play an important role in gonadal development and steroidogenesis [5–7]. This study identifies a homozygous *WWOX* c.421G > A (p.Ala141Thr) variant in a 7-month-old male presenting with hypospadias and undescended testes, providing evidence that *WWOX* may play a previously underrecognized role in the pathogenesis of 46, XY DSD. The variant, absent in 100 healthy pediatric controls, was associated with markedly reduced WWOX protein expression in both the proband and his father, as demonstrated by Western blot analysis. Supported by *in-silico* predictions of altered protein structure, these results suggest that the p.Ala141Thr substitution impairs protein stability and function, leading to disrupted gonadal differentiation. The father refused a genital exam and said his testicles and penis were all normal. However, in vitro fertilization, the absence of a uterine-ovarian problem in the mother, and the consideration of a male factor suggest the presence of 46XY DSD in the father.

Given the high expression of *WWOX* in Leydig and Sertoli cells during fetal and early postnatal testicular development, this substitution may interfere with *WWOX*-mediated redox signaling or WW–domain–dependent protein interactions critical for regulating steroidogenic enzymes.

Specifically, *WWOX* interacts with transcription factors such as p73 and steroidogenic factor-1 (SF1) (*NR5A1*), which regulate the expression of genes essential for testicular differentiation. Disruption of these pathways could impair androgen biosynthesis or Sertoli cell maturation, contributing to the observed phenotype of hypospadias and partial androgen responsiveness.

Recent findings by Wu et al. provide indirect yet compelling support for the potential role of *WWOX* in sex development [8]. Their study on *SRY*-negative pigs demonstrated that sexual differentiation can proceed through alternative genomic pathways independent of the canonical *SRY–SOX9* axis. This observation reinforces the concept that sex determination is a polygenic and network-driven process, rather than a single-gene cascade [8]. In this context, *WWOX*—given its high expression in gonadal tissues and established roles in differentiation and steroidogenesis—may function as a regulatory component within these alternative pathways, influencing gonadal development even outside the traditional sex-determining framework. In agreement with this notion, Fabbri-Scallet et al. further illustrated that non-coding variants—specifically within *NR5A1*—can alter transcriptional regulation and lead to diverse 46,XY DSD phenotypes, even in the absence of exonic mutations [9]. Together, these studies emphasize that both coding and non-coding disruptions can converge on similar developmental outcomes. Consistent with this model, our identification of a coding variant in *WWOX* that reduces protein expression supports the view that *WWOX* may act as a key structural component within this broader regulatory network, linking transcriptional control and gonadal differentiation through interdependent genomic mechanisms.

Previous research provides further support for the involvement of *WWOX* and related genetic mechanisms in disorders of sex development and broader developmental regulation. Piard et al. expanded the clinical and molecular spectrum of *WWOX*-associated disorders by reporting twenty additional cases of *WWOX*-related epileptic encephalopathy (WOREE syndrome), demonstrating that *WWOX* dysfunction can lead to combined neurological and developmental abnormalities, underscoring its pleiotropic biological roles [10]. Kim et al. demonstrated that, even with advanced targeted gene panel sequencing, approximately half of patients with severe 46,XY DSD remain without a defined genetic cause, underscoring the need to explore novel or underrecognized genes, such as *WWOX* [11]*.* In direct alignment with our findings, White et al. (12) identified a multi-exon deletion within *WWOX* in a 46,XY DSD patient presenting with ambiguous genitalia, providing the first genetic evidence that *WWOX* disruption can directly contribute to abnormal gonadal differentiation. Collectively, these studies strengthen the hypothesis that *WWOX* is a functionally significant component of the genetic network underlying gonadal development and sexual differentiation.

From a clinical perspective, identifying such variants is crucial for improving molecular diagnosis in patients with atypical 46, XY DSD, especially those with negative results in common DSD gene panels. Integrating functional assays, such as protein quantification and in silico structural prediction, can help reclassify variants of uncertain significance (VUS) and provide more accurate genetic counseling for affected families.

Our findings, therefore, highlight *WWOX* as a promising candidate gene in the differential diagnosis of DSDs and underscore the need to include it in next-generation sequencing panels for patients with unexplained 46,XY phenotypes.

A notable limitation of this study is its single-case design, which naturally narrows the generalizability of the findings across diverse genetic backgrounds. Additionally, functional validation was limited to Western blot analysis; more detailed investigations, such as cell-based assays, immunohistochemistry, or CRISPR-based modeling, will be required to confirm the mechanistic link between the *WWOX* variant and testicular development.

Furthermore, although in silico tools predicted a deleterious impact, the discordance between PolyPhen (“probably damaging”) and SIFT (“tolerated”) highlights the inherent uncertainty of computational predictions.

Future studies integrating transcriptomic profiling, hormonal phenotyping, and animal models could elucidate whether *WWOX* variants contribute to a broader class of gonadal differentiation disorders. Such research may also identify compensatory molecular pathways that could serve as therapeutic targets in cases of partial gonadal dysgenesis or hypospadias.

In summary, this study contributes to the growing body of evidence that *WWOX* plays a key role in gonadal development. The identification of the p.Ala141Thr variant and its functional consequences suggests that subtle *WWOX* perturbations may influence the delicate balance of testicular differentiation and androgen synthesis. Incorporating functional evidence alongside genetic data could transform how variants in underexplored genes, such as *WWOX*, are interpreted in the context of sex development disorders.

## Data Availability

If needed, data from this study are available upon reasonable request.
